# Oscillometric blood pressure by age and height for non overweight children and adolescents in Lubumbashi, Democratic Republic of Congo

**DOI:** 10.1186/s12872-018-0741-4

**Published:** 2018-01-19

**Authors:** Emmanuel Kiyana Muyumba, Dophra Ngoy Nkulu, Clarence Kaut Mukeng, Jacques Mbaz Musung, Placide Kambola Kakoma, Christian Ngama Kakisingi, Oscar Numbi Luboya, Françoise Kaj Malonga, Justin Kalungwe Kizonde, Olivier Mukuku, Weili Yan

**Affiliations:** 1 Department of Internal Medicine, Sendwe Hospital, University of Lubumbashi, Lubumbashi, Democratic Republic of Congo; 2 Department of Public Health, University of Lubumbashi, Lubumbashi, Democratic Republic of Congo; 3 Department of Internal Medicine, University Clinic, University of Lubumbashi, Lubumbashi, Democratic Republic of Congo; 4 Department of Pediatrics, University Clinic, University of Lubumbashi, Lubumbashi, Democratic Republic of Congo; 5 School of Public Health, University of Lubumbashi, Lubumbashi, Democratic Republic of Congo; 6 Department of Research, Higher Institute of Medical Techniques, Lubumbashi, Democratic Republic of Congo; 7 Department of Gynecology, Clinical University of Lubumbashi, University of Lubumbashi, Lubumbashi, Democratic Republic of Congo; 8 Department of Clinical Epidemiology, Children’s Hospital of Fudan University, 399 Wanyuan Road, Shanghai, 201102 China

**Keywords:** Blood pressure, Children, Adolescents, Lubumbashi, Percentile tables, GMLSS, LMS

## Abstract

**Background:**

The diagnosis of hypertension in children is complex because based on normative values by sex, age and height, and these values vary depending on the environment. Available BP references used, because of the absence of local data, do not correspond to our pediatric population. Accordingly, our study aimed to provide the BP threshold for children and adolescents in Lubumbashi (DRC) and to compare them with German (KIGGS study), Polish (OLAF study) and Chinese (CHNS study) references.

**Methods:**

We conducted a cross-sectional study among 7523 school-children aged 3 to 17 years. The standardized BP measurements were obtained using a validated oscillometric device (Datascope Accutor Plus). After excluding overweight and obese subjects according to the IOTF definition (*n* = 640), gender-specific SBP and DBP percentiles, which simultaneously accounted for age and height by using an extension of the LMS method, namely GAMLSS, were tabulated.

**Results:**

The 50th, 90th and 95th percentiles of SBP and DBP for 3373 boys and 3510 girls were tabulated simultaneously by age and height (5th, 25th, 50th, 75th and 95th height percentile).

Before 13 years the 50th and 90th percentiles of SBP for boys were higher compared with those of KIGGS and OLAF, and after they became lower: the difference for adolescents aged 17 years was respectively 8 mmHg (KIGGS) and 4 mmHg (OLAF). Concerning girls, the SBP 50th percentile was close to that of OLAF and KIGGS studies with differences that did not exceed 3 mmHg; whereas the 90th percentile of girls at different ages was high. Our oscillometric 50th and 90th percentiles of SBP and DBP were very high compared to referential ausculatory percentiles of the CHNS study respectively for boys from 8 to 14 mmHg and 7 to 13 mmHg; and for girls from 10 to 16 mmHg and 11 to 16 mmHg.

**Conclusions:**

The proposed BP thresholds percentiles enable early detection and treatment of children and adolescents with high BP and develop a local program of health promotion in schools and family.

**Electronic supplementary material:**

The online version of this article (10.1186/s12872-018-0741-4) contains supplementary material, which is available to authorized users.

## Background

Several publications have shown the importance of measuring the blood pressure (BP) and hypertension in childhood and adolescence [1–4]. In children and adolescents, high BP values are associated with left ventricular hypertrophy [5], increase in thickness intima-media of arteries [6] and they are predictive of hypertension in adulthood [7].

Pediatricians have at their disposal BP references tables, which determine whether BP is normal or if it is a threshold that requires the application of the assessment, prevention or treatment. BP references are based on sex, age and height, but also on study populations characteristics such as ethnicity or nationality, including the type of device used to measure BP [1, 2, 8, 9].

Obesity has become epidemic in the world in adults as well as in children and adolescents [10, 11]. Adiposity in childhood, as measured by body mass index (BMI) [12] is an important predictor of elevated BP. In a study among 197,191 children aged 7–17 years obtained from a Chinese national survey in 2010, Dong et al. [13] noted that overweight and obese children have a significantly higher risk of high BP than non-overweight children.

BP references or standards are not available for Congolese children in the Democratic Republic of Congo (DRC) but there are many others [1, 2, 14–16] that do not correspond to our pediatric population.

The objectives of our study were to establish the BP threshold percentiles of non-overweight children and adolescents in Lubumbashi (DRC) and compare them with German, Polish and Chinese BP references.

## Methods

### Design, participants and setting of the study

The study took place in Lubumbashi (Province of Haut-Katanga) the second city of the DRC by its economic, political, social and cultural importance. The studied population was composed of school children aged 3 to 17 years, enrolled in pre-primary, primary and secondary schools during the school-years 2013–2014, 2014–2015 and 2015–2016.

A cross-sectional study was conducted on a representative sample of these pupils, who were recruited in randomly selected clusters using two-stage sampling. The first cluster was comprised of selected schools from all private and public schools of Lubumbashi. The second cluster was comprised of randomly selected classes within each of the selected schools. All pupils in the selected classes were invited to join the study. Sampling was stratified by township.

For a strate (township), the equation allowing the calculation of the sample was the following:

$$ n={z}^2\frac{p\left(1-p\right)}{e^2} $$

In which: - z is the level of confidence (= 95%).

- *p* is the initial level of the school attendance rate (= 50%)*.*

- *e* is the error margin (= 0.05%)*.*

The size calculated in this way had been adjusted according to the effect of the sampling plan (= 1.5%), the number of estimations by age group and by sex (= 3%) and this was reported to the expected number of non-answers (= 20%). The number thus obtained was multiplied by the number of the strate (township) of the city of Lubumbashi (for details, please consult website www.unilu.ac.cd/wp-content/uploads/2016/07/Seuils1.pdf).

Informed written consent was obtained from parents or guardians. The approval to conduct the study and authorizations were obtained from the Medical Ethic Committee of the University of Lubumbashi (UNILU/CEM/027/2013–27 september 2013), the Provincial Ministry of Education, Scientific Research, Transport and Energy of Katanga (N° 10.5/0209/CAB/MIN.PROV/ED.R.TE/KAT/2014–11 march 2014) and the authorities of the selected schools.

Data were collected by trained medical staff. The study team received refresher training at the beginning of each data collection phase.

### BP measurement

BP was measured by using a Datascope Accutorr Plus (Datascope Corporation, USA). The appropriate cuff size (bladder width at least 40% of arm circumference and length to cover 80–100% of arm circumference) was determined by measuring the mid-upper arm circumference.

BP measurements were performed at least 30 min after exercise or the last meal, in a subject at rest 5 min before setting, in a seated position with arm and back supported, feet resting on the floor and legs uncrossed. The cuff was applied to the right arm, at the heart level, then wrapped in a sealing which did not allow two fingers to be inserted under it. The lower edge of the cuff was placed 2 cm from the cubital fossa. Three readings were obtained at a 1 min intervals on the same day and the mean of the second and third readings was used for analysis.

### Anthropometric measurements

Body weight was measured in duplicate with the participant wearing light underwear, barefoot, standing on a digital scale. Body weight was recorded nearest 0.1 kg. Body height recorded nearest 0.1 cm was measured in duplicate. Each participant was in standing upright position, barefoot with shoulders and hips perpendicular at the central axis, heels against the step, knees together, arms relaxed along the body and head straight. Special attention was given to children (under 6 years) using a second investigator to block the movement of the knees. The mean of the two measurements of weight and height and were selected for statistical analysis.

BMI was calculated as weight (in kilograms) divided by the square of height (in meters). The terms overweight and obese were defined according to the International Obesity Task Force (IOTF) definition [17].

### Inclusion and exclusion criteria for the sample on which the percentiles are based

Of 8371 participants consented, we excluded 1488: 795 children were younger than 3 years (*n* = 55) or over 17 years (*n* = 740), 53 had outlying or missing data (date of birth, BP, weight, height) and 640 were recorded overweight (*n* = 548) or obese (*n* = 92). No child had been reported to have a chronic disease likely to influence weight or blood pressure and also no child was taking a medicine having an influence on the blood pressure (Additional file 1).

### Statistical analysis

All statistical analyses were performed using SPSS version 22.0 (SPSS Inc., Chicago, IL, USA) and the free statistical software R 3.1.2 (2014–10-31) (http://www.cran.r-project.org).

Thresholds of BP by gender were constructed by age and height simultaneously using an extension of the LMS method [18], namely generalized additive models for location scale and shape (GAMLSS) with the Box-Cox power exponential (BCPE) and Box-Cox-Cole-Green (BCCG) distributions families, fitted with GAMLSS 4.3–1 in the free statistical software R 3.1.2 (2014–10-31). In GAMLSS [18, 19], four parameters (μ, σ, ν, τ) were used to define the location, scale and shape of the BP distribution with age and height. To obtain the optimal models by minimizing the Schwarz Bayesian Criterion [20], linear and additive effect of age and height on systolic blood pressure (SBP) and diastolic blood pressure (DBP) were modeled simultaneously. The threshold values of the 50th, 90th and 95th percentiles of SBP and DBP were calculated by age and height (exact heights according to the 5th, 25th, 50th, 75th and 95th percentiles) for boys and girls separately.

Different percentiles of SBP and DBP (50th and 90th percentiles) for boys and girls, were compared with references percentiles of the German Health Interview and Examination Survey for Children and Adolescents (KIGGS) [14], the Elaboration of the Ranks of Reference Arterial Blood Pressure for the Population of Children and Adolescents in Poland (OLAF) [15] and the Chinese reference that used data from the China Health and Nutrition Survey (CHNS) [16]. This comparison was made for the different ages in relation to the target population of these studies (3–17 years for KIGGS and 7–17 years for OLAF and CHNS).

## Results

Of the 11,283 pupils selected and invited to take part in the study, 8371 children and adolescents consented and were enrolled for a global participation rate of 74.2%. Data were collected three times (according to the school calendar in the DRC) from March 2014 to December 2015; in 66 schools. The reference population of non-overweight children and adolescents aged 3 to 17 years consisted of 3373 boys and 3510 girls.

Table 1 summarized the baseline characteristics of 6883 non-overweight children and adolescents. The mean of SBP and DBP increased with age in both sexes. The mean of the first BP (both systolic and diastolic) was the highest and the mean of the third BP was the lowest in the series of three measures in both sexes and in all age groups. The mean of the first and second BP was higher: 0.8 to 1.4 mmHg SBP and 0.9 to 2.0 mmHg DBP, in comparison with the average of the second and third BP (Table 1).

**Table 1:** Baseline characteristics of children and adolescents of normal weight aged 3 to 17 years in Lubumbashi, DRC

	Age, year			
	3–6	7–10	11–13	14–17
n, included (% 3373 boys, 3510 girls)				
Boys	480 (14.2)	1161 (34.4)	995 (29.5)	737 (21.9)
Girls	393 (11.2)	1133 (32.3)	1014 (28.9)	970 (27.6)
Weight, mean (SD), kg				
Boys	18.7 (3.1)	29.7 (4.8)	35.9 (6.5)	50.9 (8.8)
Girls	18.6 (3.5)	27.1 (5.5)	39.0 (7.5)	50.4 (6.5)
Height, median (SD), cm				
Boys	111.1 (8.8)	130.5 (9.0)	142.3 (9.4)	162.3 (10.2)
Girls	111.3 (10.2)	131.2 (9.9)	148.0 (9.6)	159.0 (7.4)
IMC, mean (SD) kg / m^2^				
Boys	15.1 (1.2)	15.6 (1.4)	16.9 (1.7)	19.2 (2.1)
Girls	14.9 (1.1)	15.6 (1.5)	17.7 (2.1)	19.9 (2.1)
First SBP, mean (SD), mmHg				
Boys	101.7 (11.0)	104.6 (10.6)	109.2 (10.3)	118.0 (12.4)
Girls	102.0 (11.1)	106.7 (10.7)	112.6 (11.9)	116.9 (11.3)
Second SBP, mean (SD), mmHg				
Boys	100.7 (11.0)	103.4 (10.7)	107.7 (10.5)	116.7 (12.3)
Girls	100.3 (10.6)	105.5 (10.7)	111.2 (11.1)	115.5 (10.8)
Third SBP, mean (SD), mmHg				
Boys	99.7 (10.9)	102.4 (10.0)	106.8 (9.9)	115.3 (11.9)
Girls	99.4 (10.3)	104.1 (10.2)	110.1 (10.8)	114.1 (10.6)
Mean of first and second SBP, mean (SD), mmHg				
Boys	101.2 (10.4)	104.0 (9.9)	108.5 (9.7)	117.4 (11.7)
Girls	101.2 (10.2)	106.1 (10.1)	111.9 (10.8)	116.2 (10.5)
Mean of second and third SBP, mean (SD), mmHg				
Boys	100.2 (10.4)	102.9 (9.8)	107.2 (9.7)	116.0 (11.6)
Girls	99.8 (9.8)	104.8 (10.0)	110.6 (10.4)	114.8 (10.2)
First DBP, mean (SD), mmHg				
Boys	62.8 (9.6)	65.1 (9.1)	67.1 (7.9)	69.4 (9.0)
Girls	63.5 (9.5)	66.2 (9.2)	68.2 (8.8)	70.5 (8.5)
Second SBP, mean (SD), mmHg				
Boys	61.8 (8.5)	63.4 (9.0)	65.4 (8.4)	67.5 (8.8)
Girls	61.9 (9.6)	65.0 (8.7)	66.4 (8.7)	68.1 (8.5)
Third DBP, mean (SD), mmHg				
Boys	61.0 (8.9)	62.2 (8.9)	64.2 (8.1)	67.0 (9.2)
Girls	60.9 (8.8)	63.7 (8.9)	65.2 (9.0)	67.0 (8.5)
Mean of first and second DBP, mean (SD), mmHg				
Boys	62.3 (8.0)	64.3 (8.0)	66.3 (7.2)	68.4 (7.9)
Girls	62.7 (8.5)	65.8 (7.8)	67.3 (7.7)	69.3 (7.6)
Mean of second and third DBP, mean (SD), mmHg				
Boys	61.4 (7.7)	62.8 (7.9)	64.8 (7.4)	67.3 (8.1)
Girls	61.4 (8.1)	64.3 (7.8)	65.8 (7.9)	67.6 (7.6)

*SD* standard deviation, *BMI* body mass index, *SBP* systolic blood pressure, *DBP* diastolic blood pressure

Among the best fitted models for the 4 parameters of distribution of SBP and DBP, the Box-Cox and Cole Green (BCCG) was shown as the best fit model of SBP for both genders and DBP for girls; while the Box-Cox Power Exponential (BCPE) was for the boys’ DBP.

The thresholds 50th, 90th and 95th percentiles of SBP and DBP were tabulated simultaneously by age and exact height (5th, 25th, 50th, 75th and 95th height percentile) respectively for boys and girls and are shown in Tables 2 (for boys) and 3 (for girls). The BP increased both in relation to age and height in both sexes. The median SBP and DBP were higher (about 2 mmHg) in girls up to the age 14 years, after this age they were almost similar in both sexes. As an illustration, in adolescents aged 17 years with a median height (167 cm for boys and 162 cm for girls), the median percentiles of SBP and DBP were similar, respectively 115 mmHg and 68 mmHg. The 95 percentile used to define hypertension in children and adolescents had varied between the 5th and 95th percentile height (SBP in boys: 4–7 mmHg and in girls: 5–8 mmHg; DBP in boys: 1–3 mmHg and in girls: 1–2 mmHg).

**Table 2:** Age-height-specific thresholds: 50th, 90th and 95th percentiles of SBP and DBP values for boys aged 3–17 years

Age, year	Height, cm	Height percentile	SBP, mmHg				DBP, mmHg			
			S*	50th percentile (median)	90th percentile	95th percentile	S*	50th percentile (median)	90th percentile	95th percentile
3	87	5th	0.0983	91	103	107	0.0712	59	68	71
	94	25th	0.0974	92	105	109	0.0678	59	69	72
	97	50th	0.0970	93	105	110	0.0667	59	69	72
	101	75th	0.0964	94	106	111	0.0647	60	69	72
	107	95th	0.0956	95	108	112	0.0623	60	70	72
4	95	5th	0.0972	93	106	110	0.0674	60	69	72
	99	25th	0.0967	94	107	111	0.0655	60	70	72
	103	50th	0.0961	95	108	112	0.0638	60	70	73
	108	75th	0.0955	96	108	113	0.0619	60	70	73
	112	95th	0.0949	97	110	114	0.0599	61	70	73
5	101	5th	0.0964	95	108	112	0.0648	60	70	73
	106	25th	0.0957	96	109	113	0.0626	61	70	73
	109	50th	0.0953	97	109	114	0.0612	61	71	73
	114	75th	0.0946	98	111	115	0.0592	61	71	74
	122	95th	0.0936	99	112	116	0.0561	61	71	74
6	105	5th	0.0959	96	109	113	0.0630	61	71	73
	112	25th	0.0949	98	111	115	0.0601	61	71	74
	116	50th	0.0944	98	112	116	0.0584	61	71	74
	120	75th	0.0938	99	113	117	0.0567	62	72	74
	127	95th	0.0929	101	114	118	0.0542	62	72	75
7	112	5th	0.0949	98	111	116	0.0598	62	71	74
	118	25th	0.0941	99	113	117	0.0576	62	72	75
	123	50th	0.0935	100	114	118	0.0557	62	72	75
	126	75th	0.0930	101	114	119	0.0544	62	72	75
	137	95th	0.0916	103	117	121	0.0506	63	73	76
8	112	5th	0.0949	99	112	116	0.0599	62	72	75
	123	25th	0.0934	101	114	119	0.0556	62	72	75
	128	50th	0.0928	102	115	120	0.0538	63	73	76
	132	75th	0.0922	103	116	121	0.0523	63	73	76
	140	95th	0.0912	105	118	122	0.0495	63	74	77
9	122	5th	0.0936	101	115	119	0.0562	63	73	76
	127	25th	0.0930	102	116	120	0.0543	63	73	76
	132	50th	0.0923	103	117	121	0.0525	63	73	76
	137	75th	0.0917	104	118	122	0.0507	63	74	77
	145	95th	0.0906	106	120	124	0.0479	64	74	77
10	123	5th	0.0934	102	115	120	0.0556	63	73	76
	131	25th	0.0924	104	117	121	0.0527	64	74	77
	136	50th	0.0917	105	118	123	0.0509	64	74	77
	141	75th	0.0910	106	119	124	0.0491	64	74	77
	149	95th	0.0901	108	121	125	0.0466	64	75	78
11	129	5th	0.0927	104	117	122	0.0536	64	74	77
	136	25th	0.0917	105	119	123	0.0508	64	74	77
	142	50th	0.0910	107	120	124	0.0489	64	75	78
	147	75th	0.0903	108	121	126	0.0471	65	75	78
	156	95th	0.0892	110	123	128	0.0443	65	76	79
12	132	5th	0.0922	105	119	123	0.0522	64	74	77
	140	25th	0.0912	107	120	125	0.0495	65	75	78
	145	50th	0.0906	108	121	126	0.0479	65	75	78
	151	75th	0.0898	109	123	127	0.0460	65	76	79
	161	95th	0.0886	111	125	129	0.0430	66	76	79
13	133	5th	0.0921	106	119	124	0.0519	65	75	78
	143	25th	0.0909	108	121	126	0.0487	65	75	78
	150	50th	0.0900	109	123	127	0.0464	65	76	79
	157	75th	0.0890	111	125	129	0.0440	66	76	79
	164	95th	0.0882	112	126	131	0.0420	66	77	80
14	139	5th	0.0914	108	121	126	0.0499	65	75	78
	149	25th	0.0901	110	123	128	0.0466	66	76	79
	157	50th	0.0891	111	125	130	0.0442	66	77	80
	164	75th	0.0883	113	127	131	0.0422	66	77	80
	172	95th	0.0872	115	128	133	0.0398	67	78	81
15	145	5th	0.0906	109	123	128	0.0479	66	76	79
	157	25th	0.0891	112	126	130	0.0442	67	77	80
	162	50th	0.0884	113	127	131	0.0425	67	77	81
	168	75th	0.0876	114	128	133	0.0408	67	78	81
	177	95th	0.0866	116	130	135	0.0385	67	78	82
16	153	5th	0.0896	112	126	130	0.0453	67	77	80
	161	25th	0.0886	113	127	132	0.0430	67	78	81
	168	50th	0.0877	115	129	133	0.0410	67	78	81
	172	75th	0.0871	116	130	134	0.0397	68	78	82
	179	95th	0.0864	117	131	136	0.0380	68	79	82
17	153	5th	0.0895	112	126	131	0.0452	67	77	81
	165	25th	0.0881	115	129	133	0.0419	68	78	81
	167	50th	0.0878	115	129	134	0.0411	68	78	82
	173	75th	0.0871	116	130	135	0.0395	68	79	82
	180	95th	0.0862	118	132	136	0.0377	68	79	82

*SBP* systolic blood pressure, *DBP* diastolic blood pressure

S *, coefficient of variation of blood pressure

**Table 3:** Age-height-specific thresholds: 50th, 90th and 95th percentiles of SBP and DBP values for girls aged 3–17 years

Age, year	Height, cm	Height percentile	SBP, mmHg				DBP, mmHg			
			S *	50th percentile (median)	90th percentile	95th percentile	S *	50th percentile (median)	90th percentile	95th percentile
3	88	5th	0.0994	93	106	109	0.1313	60	70	73
	92	25th	0.0996	94	107	110	0.1313	60	70	74
	99	50th	0.0998	95	108	112	0.1313	60	71	74
	103	75th	0.1000	96	109	113	0.1313	60	71	74
	109	95th	0.1002	97	110	114	0.1313	61	71	75
4	95	5th	0.0984	95	108	111	0.1298	60	71	74
	98	25th	0.0985	96	108	112	0.1298	61	71	74
	102	50th	0.0987	97	109	113	0.1298	61	71	74
	108	75th	0.0989	98	111	114	0.1298	61	72	75
	121	95th	0.0993	100	113	117	0.1298	62	72	76
5	98	5th	0.0973	96	109	113	0.1283	61	71	74
	105	25th	0.0975	98	110	114	0.1283	61	72	75
	110	50th	0.0977	98	111	115	0.1283	61	72	75
	115	75th	0.0979	99	113	117	0.1283	62	72	75
	122	95th	0.0981	101	114	118	0.1283	62	73	76
6	105	5th	0.0963	98	111	115	0.1269	62	72	75
	113	25th	0.0966	100	113	117	0.1269	62	72	76
	117	50th	0.0967	100	114	118	0.1269	62	73	76
	123	75th	0.0969	101	115	119	0.1269	62	73	76
	131	95th	0.0972	103	117	121	0.1269	63	73	77
7	110	5th	0.0953	100	113	116	0.1254	62	73	76
	118	25th	0.0955	101	114	118	0.1254	62	73	76
	123	50th	0.0957	102	116	120	0.1254	63	73	76
	128	75th	0.0959	103	116	121	0.1254	63	73	77
	134	95th	0.0961	104	118	122	0.1254	63	74	77
8	117	5th	0.0943	102	115	118	0.1240	63	73	76
	123	25th	0.0945	103	116	120	0.1240	63	74	77
	128	50th	0.0947	104	117	121	0.1240	63	74	77
	133	75th	0.0948	104	118	122	0.1240	63	74	77
	142	95th	0.0952	106	120	124	0.1240	64	75	78
9	121	5th	0.0932	103	116	120	0.1226	63	74	77
	129	25th	0.0935	104	118	122	0.1226	64	74	77
	133	50th	0.0937	105	119	123	0.1226	64	74	77
	138	75th	0.0938	106	120	124	0.1226	64	75	78
	149	95th	0.0942	108	122	127	0.1226	65	75	78
10	126	5th	0.0922	104	118	122	0.1212	64	74	77
	133	25th	0.0925	106	119	123	0.1212	64	75	78
	137	50th	0.0926	107	120	124	0.1212	64	75	78
	143	75th	0.0928	108	121	126	0.1212	65	75	78
	152	95th	0.0931	109	123	128	0.1212	65	76	79
11	127	5th	0.0911	105	118	122	0.1198	64	75	78
	138	25th	0.0915	107	121	125	0.1198	65	75	78
	145	50th	0.0917	108	122	127	0.1198	65	76	79
	150	75th	0.0919	110	123	128	0.1198	65	76	79
	157	95th	0.0921	111	125	130	0.1198	66	76	79
12	132	5th	0.0901	107	120	124	0.1184	65	75	78
	142	25th	0.0905	109	122	126	0.1184	65	76	79
	149	50th	0.0907	110	124	128	0.1184	66	76	79
	154	75th	0.0908	111	125	129	0.1184	66	76	79
	161	95th	0.0911	112	126	131	0.1184	66	77	80
13	139	5th	0.0892	109	122	126	0.1171	65	76	79
	148	25th	0.0895	110	124	128	0.1171	66	76	79
	154	50th	0.0897	111	125	130	0.1171	66	77	80
	159	75th	0.0899	112	126	131	0.1171	66	77	80
	166	95th	0.0901	114	128	133	0.1171	67	77	80
14	143	5th	0.0882	110	123	128	0.1157	66	76	79
	153	25th	0.0885	112	125	130	0.1157	66	77	80
	157	50th	0.0887	113	126	131	0.1157	67	77	80
	162	75th	0.0888	113	128	132	0.1157	67	77	80
	168	95th	0.0890	115	129	134	0.1157	67	78	81
15	147	5th	0.0872	111	125	129	0.1144	67	77	80
	154	25th	0.0874	113	126	131	0.1144	67	77	80
	159	50th	0.0876	113	127	132	0.1144	67	77	80
	164	75th	0.0878	114	128	133	0.1144	67	78	81
	171	95th	0.0880	116	130	135	0.1144	68	78	81
16	148	5th	0.0862	112	125	130	0.1131	67	77	80
	156	25th	0.0864	114	127	131	0.1131	67	77	80
	161	50th	0.0865	114	128	133	0.1131	68	78	81
	164	75th	0.0867	115	129	133	0.1131	68	78	81
	174	95th	0.0870	117	131	136	0.1131	68	78	81
17	150	5th	0.0851	113	126	131	0.1118	67	77	80
	156	25th	0.0853	114	128	132	0.1118	68	78	81
	162	50th	0.0855	115	129	133	0.1118	68	78	81
	166	75th	0.0856	116	130	134	0.1118	68	78	81
	173	95th	0.0858	117	131	136	0.1118	68	79	82

*SBP* systolic blood pressure, *DBP* diastolic blood pressure

S *, coefficient of variation of blood pressure

For adolescents boys aged 11–17 years and girls aged 10–17 years, the 90th SBP percentile for the median height was respectively 120–132 mmHg and 120–131 mmHg.

**Fig. 1:**
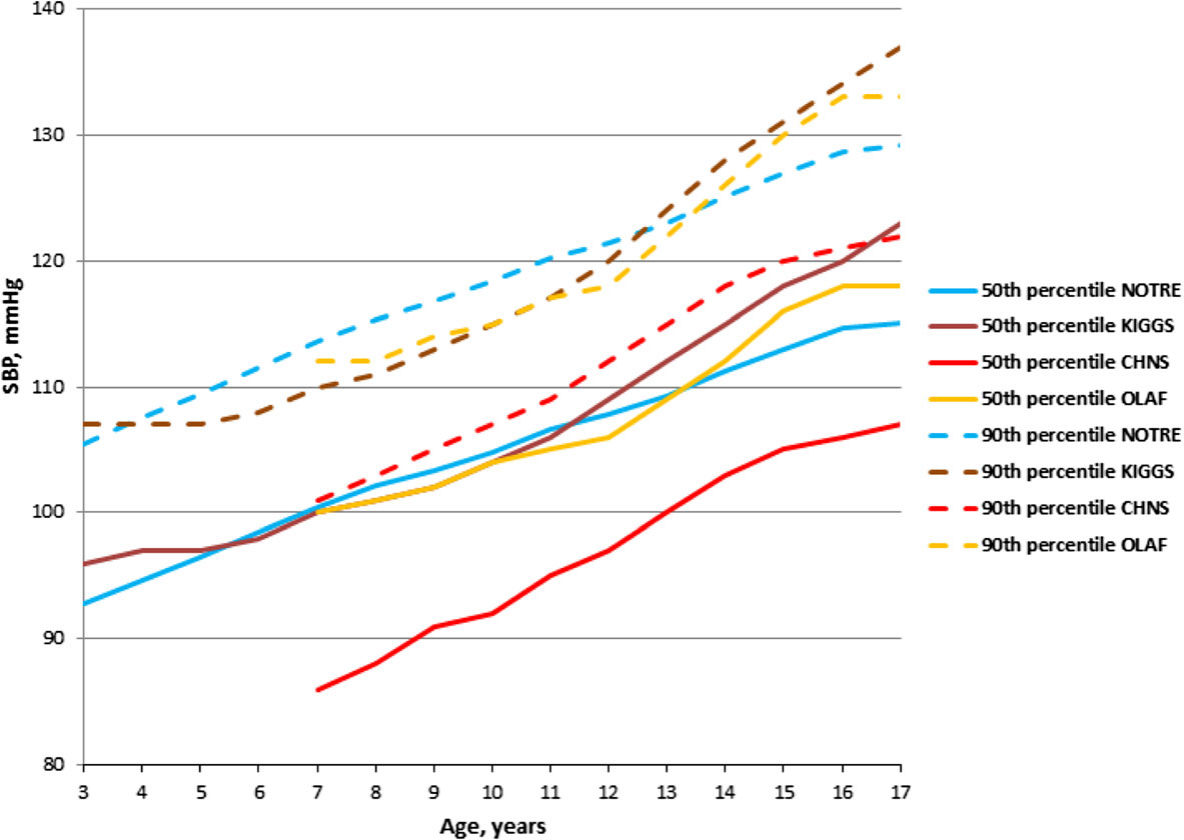
The 50th and 90th percentiles of SBP for the median height for our study (NOTRE) compared with the German (KIGGS), Polish (OLAF) and Chinese (CHNS) boys

**Fig. 2:**
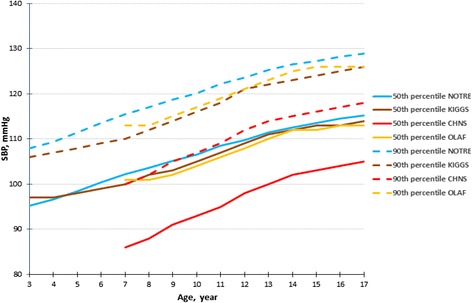
The 50th and 90th percentiles of SBP for the median height for our study (NOTRE) compared with the German (KIGGS), Polish (OLAF) and Chinese (CHNS) girls

In the case of boys aged 5–12 years, the 50th SBP percentile of our oscillometric threshold was similar to the corresponding percentile of German oscillometric reference (KIGGS study), whereas compared with the Polish oscillometric reference (OLAF study) the difference were less than 2 mmHg in age 7, 8 and 13, 14 years (Fig. 1). The 50th percentile of SBP of the KIGGS study was higher for boys >14 years (8 mmHg for adolescents aged 17 years). With regard to girls, the 50th SBP percentile of our study was close to the corresponding percentile of the KIGGS and OLAF studies: the differences did not exceed 2 mmHg, except among girls aged 9 and 10 years (for OLAF study) in which the difference was greater than 3 mmHg (Fig. 2).

For boys <13 years the 90th SBP percentile was higher from 1 to 4 mmHg in comparison to KIGGS and OLAF percentiles. After, it became progressively lower: for adolescents aged 17 the difference was 8 mmHg with the KIGGS study and 4 mmHg with the OLAF study (Fig. 1). In the case of girls, our 90th SBP percentile was consistently higher compared to KIGGS and OLAF percentiles (Fig. 2).

**Fig. 3:**
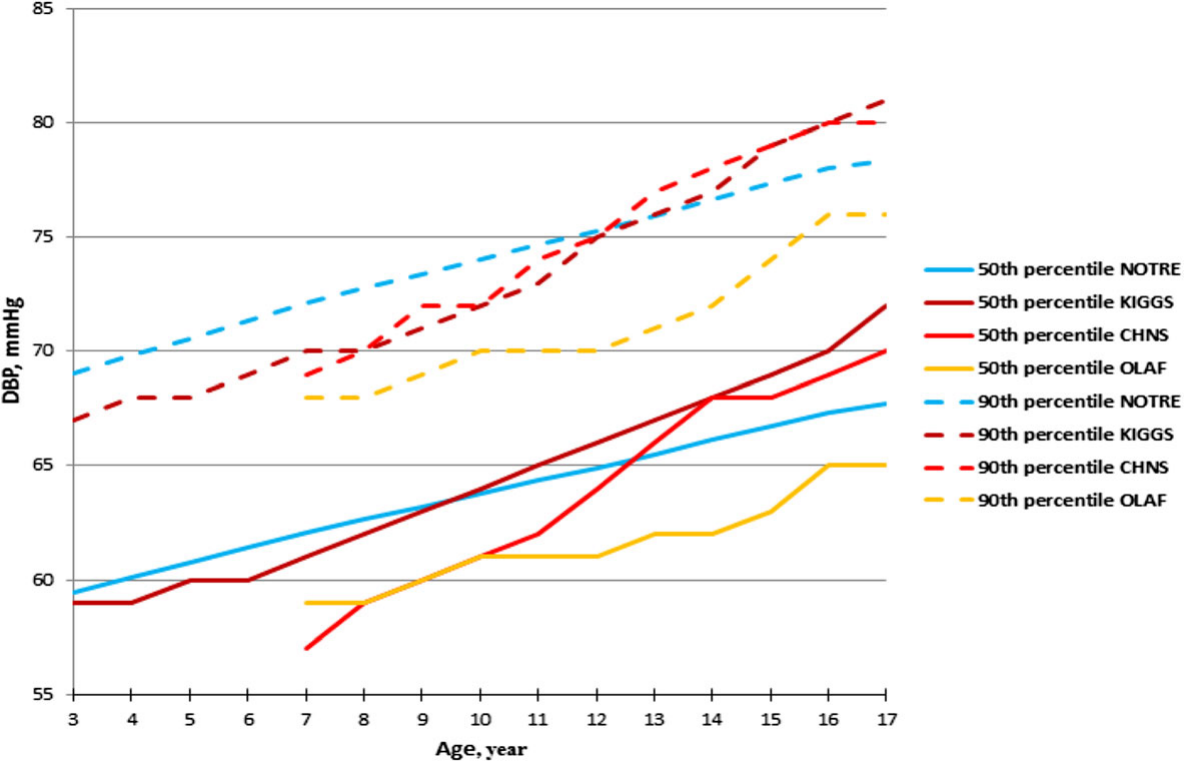
The 50th and 90th percentiles of DBP for the median height for our study (NOTRE) compared with the German (KIGGS), Polish (OLAF) and Chinese (CHNS) boys

The 50th and 90th DBP percentiles of the KIGGS boys (Fig. 3) were higher at all ages (among adolescents aged 17 years the difference was 4 mmHg). In case of the girls age range 7–17 years, the 50th and 90th DBP percentiles of OLAF study were higher when compared with our corresponding percentile.

**Fig. 4:**
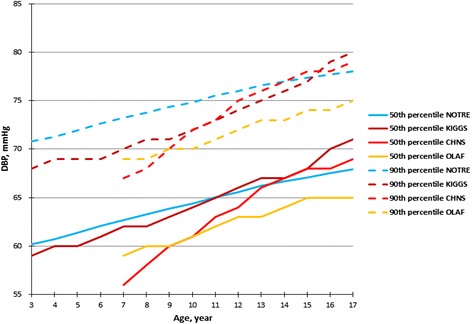
The 50th and 90th percentiles of DBP for the median height for our study (NOTRE) compared with the German (KIGGS), Polish (OLAF) and Chinese (CHNS) girls

In comparing the 50th and 90th SBP percentiles for median height for our oscillometric values to the Chinese ausculatory referential (CHNS) values, the 50th and 90th percentiles were consistently very higher among the children and adolescents of the same age: the difference was 8 to 14 mmHg and 7 to 13 mmHg for boys (Fig. 1); and 10 to 16 mmHg and 11–16 mmHg for girls (Fig. 2). The 50th and 90th DBP percentiles for boys (Fig. 3) and girls (Fig. 4) of our study were higher before age 11 years in comparison to the CHNS study; and in the age range 11–17 years, the difference were relatively minor (not exceeding 2 mmHg).

## Discussion

This study presents the first BP threshold percentiles for children and adolescents of normal weight aged 3–17 years in Lubumbashi (DRC), computed by age and height simultaneously, by using an improved statistical method named GAMLSS provided in the package of R software [14, 16, 19]. The BP percentiles were established on the basis of oscillometric measurements of the BP with a device clinically validated in children: the Datascope Accutorr Plus [21]. This device has also been used in several large studies related to BP in children and adolescents worldwide [14, 15, 22]. In addition, the oscillometric BP measurement is increasingly used in pediatric clinical practice.

We have not included overweight or obese subjectsin our study. Using a sample of normal weight to develop percentile allows our proposed BP thresholds to be more sensitive to the identification of children and adolescents with high BP because we avoided certain risk factors associated with the BP as being overweight or obese [10, 13]. Sorof et al. [23] found a strong correlation between the BP and overweight and obesity.

Our mean SBP (for girls) and DBP (for boys and girls) were higher compared to those observed by Kulaga et al. [15]. Compared to the mean of the first and second BP (both systolic and diastolic) values were higher than those of children and adolescents in the KIGGS study [14].

The height is a key covariate associated with BP levels [1]. Indeed, not taking into account the height in establishing the BP references could lead to an inaccurate assessment of the BP in pediatric practice particularly for children who are very small (5th percentile) or very tall (95th percentile). Our BP thresholds do not require the use of height reference tables because it is presented in centimeters. In addition, this presentation of exact height by value (in centimeters) in different categories of height percentile is proposed to the allow evaluation of the children and adolescents BP more convenient and accurate.

We proposed the 90th and 95th BP percentiles to allow the detection of prehypertension and hypertension in children and adolescents. These cut-off values for prehypertension and hypertension respectively, were used according to the criteria’s definition of the Fourth Report on the Diagnosis, Evaluation and Treatment of High Blood Pressure in Children and Adolescents [1] and to the recommendations of the European Society of Hypertension on the Management of High Blood Pressure in Children and Adolescents [2]. Because of the large amount of data available, the Task Force for Blood Pressure in Children [1] is still the study of reference. We have not presented the 99th BP percentile. Indeed, a child or adolescent with BP value that defines hypertension (≥ 95th percentile compared to reference tables) will not be diagnosed by this measure alone as being hypertensive but other additional BP measures are recommended on different occasions [1, 2]. Owing to cultural and ethnic diversity of the peoples of the DRC, our results cannot extrapolate to the entire nation.

The 90th SBP percentile for the median height of adolescents for boys aged 11–17 years and girls aged 10–17 years, respectively of 120–132 mmHg and 120–131 mmHg, was equal to or higher than the threshold 120 mmHg for the identification of prehypertension as recommended by the fourth report of the National High Blood Pressure Education Program (NHBPEP) Working Group on High Blood Pressure in Children and Adolescents [1], the European Society of Hypertension [2] and the Seventh Report of the Joint National Committee on Prevention, Detection, Evaluation, and treatment of High Blood Pressure (JNC VII) [24]. This observation may indicate the need for careful consideration of changes to the definition of prehypertension in the case of adolescent. The fact that the BP of Congolese children of Lubumbashi is so high at the beginning of adolescence, could be justified by several observations showing that hypertension in black subjects occurs at the early age [25, 26]. In addition, the 90th BP percentile for adolescents aged 17 years (in both sexes) was 130 mmHg, which is equal to the recommended optimal SBP for adult to define metabolic syndrome [27]. Our results are almost similar to those reported by Kulaga et al. [15] and Neuhauser et al. [14]. For the first authors, the 90th SBP percentile for adolescents age range 13–17 years (boys) and 12–17 years (girls) was respectively 122–133 mmHg and 120–126 mmHg. While for the second author, adolescents aged 12 to 17 (both boys and girls) had their 90th SBP percentile respectively 120–137 mmHg and 122–126 mmHg. In the CHNS study [16], only adolescent boys aged 15 to 17 had the 90th percentile of SBP and DBP at 122 and 80 mmHg close to the threshold of 120/80 mmHg for the identification of prehypertension. In this study the BP values are examined by mercury sphygmomanometer.

We compared our percentiles with the KIGGS [14], OLAF [15] and CHNS [16] studies because they are all tools that aim to solve the same problem: screening and detection of elevated BP in children. In addition, the construction of the BP percentiles was based on the normal weight subjects and developed simultaneously by age and height.

Several reasons could explain the differences with each study. In particular, the devices used for the BP measurement (auscultatory, oscillometric). It is known as oscillometric devices provide high values of the BP compared to mercury sphygmomanometer [1]. The Datascope Accutor Plus had passed the standards of the Association for the Advancement of Medical Instrumentation [28] and the British Hypertension Society [29] for adults and had been validated in children aged 5 to 15 years compared to the mercury sphygmomanometer as required of the International Protocol of the European Society of Hypertension (ESH-IP) [30]. In the validation study in children [21], measures of the Datascope Accutor Plus were close to the sphygmomanometer measurements: the mean (SD) of the differences was for PAS values (oscillometric least auscultation) of - 0.9 (4.33) mmHg and DBP - 1.2 (6.48) mm Hg. The number of the BP measurements used for the establishment of the BP percentiles. As for Kulaga et al. [15], our study used the mean of the last two measures (of the three) BP for statistical analysis because the first one are often high [31, 32]. The statistical method used for the BP percentiles construction. The GAMLSS method was used in our study and those of Neuhauser et al. [14] et Yan et al. [16]; while Kulaga et al. [15] had used the polynomial regression. Another possible reason is the lack of a uniform definition for overweight and obesity for the non-inclusion of this group of children and adolescents in the study of normal weight population. Neuhauser et al. [14] had used the 90th percentile of the BMI of the German reference and Yan et al. [16] were based on the reference BMI of Chinese children and adolescents. Like in the OLAF study [15], we used the definition IOTF [17], because we have not BMI reference for children in our country and it is consistent with the levels of overweight and obesity in childhood and adolescence (2–18 years), with the definition of overweight (≥ 25 kg / m2) and obesity (≥ 30 kg / m2) in adults. We also took into account the recommendations of the European Childhood Obesity Group [33]: which suggest the use of the definition of the IOTF or the WHO definition in epidemiological studies. Ethnic, racial, geographic differences may also explain the variability of the BP in the populations studied [25, 34].

A possible limitation of our study is the selection bias. Owing to the lack of official documents, we used a reported age declaration by parents or guardians. Another limitation was related to a lack external validation made to assess the performance of our proposed BP thresholds.

## Conclusion

We established, for the first time, the thresholds percentiles (50, 90 and 95) of the BP for specific age and height of children and adolescents aged 3 to 17 years in Lubumbashi (DRC) for the use in pediatric clinical practice. Early identification of prehypertension and hypertension in children and adolescents leads to early action to the support and possibly the prevention of late morbidity and mortality. The BP thresholds percentiles proposed by the current study enable to develop a local program of health promotion in schools and family.

We observed that the 90th percentile of SBP in early adolescence is high and this corresponds to the prehypertension thresholds requiring further studies.

## Additional file

Additional file 1: Data on Oscillometric Blood Pressure for Non Overweight Children and Adolescents in Lubumbashi. Tabular datasets of this study is presented as an Excel spreadsheet. (XLSX 2513 kb)

## Abbreviations

BCCE: Box-Cox and Cole Green
BCPE: Box-Cox power exponential
BMI: Body mass index
BP: Blood pressure
CHNS: China Health and Nutrition Survey
DBP: Diastolic blood pressure
DRC: Democratic Republic of Congo
ESH: European Society of Hypertension
GMLSS: Generalized Additive Models for Location, Shape, and Scale
IOTF: International Obesity Task Force
IP-ESH: International Protocol of the European Society of Hypertension
JNC VII: Seventh Report of the Joint National Committee on Prevention, Detection, Evaluation, and treatment of High Blood Pressure
KIGGS: German Health Interview and Examination Survey for Children and adolescents
LMS: Lambda Mu Sigma
NHBPEP: National High Blood Pressure Education Program Working Group on High Blood Pressure in Children and Adolescents
OLAF: Elaboration of the reference range of arterial blood pressure for the population of children and adolescents in Poland
SBP: Systolic blood pressure
SD: Standard deviation
SPSS: Statistical Package for Social Sciences
